# Knowledge, Attitudes, and Perceptions of HPV Vaccination for Adolescents with Intellectual Disabilities: A Systematic Review †

**DOI:** 10.3390/vaccines14090764

**Published:** 2026-09-01

**Authors:** Giuseppina Lo Moro, Erica De Vita, Gianluca Voglino

**Affiliations:** 1 Department of Public Health and Pediatric Sciences, University of Turin, 10126 Turin, Italy; giuseppina.lomoro@unito.it; 2 Department of Translational Research and New Technologies in Medicine, University of Pisa, 56100 Pisa, Italy; 3 Ministry of Health, 00144 Rome, Italy; gianluca.voglino@gmail.com

**Keywords:** human papillomavirus, intellectual disability, health knowledge, attitudes, practice

## Abstract

Background/Objectives: Adolescents with intellectual disabilities may experience inequities in preventive healthcare, including HPV vaccination. This review aimed to synthesize evidence on HPV vaccination-related knowledge, attitudes and perceptions among adolescents with intellectual disabilities, parents/caregivers, educators, healthcare professionals and other stakeholders. Methods: A systematic search was conducted in PubMed, Embase and Scopus on 29 October 2025. Eligible studies reported original data on knowledge, attitudes or perceptions regarding HPV vaccination for adolescents with intellectual disabilities or relevant stakeholders. The protocol was registered on PROSPERO (CRD420251015183). Quantitative, qualitative and mixed-methods studies were considered. Results: Seven studies were included. They were conducted in the USA, Australia and the UK and involved parents/caregivers, healthcare providers, school staff, immunization staff and adolescents or young people with disabilities. Direct evidence from adolescents was limited. HPV-related knowledge was often limited or uneven, and standard information materials were perceived as insufficiently accessible. Parents/caregivers were often broadly supportive of vaccination, but HPV-specific acceptance was affected by perceived low susceptibility, assumptions about sexual inactivity, safety concerns, procedure-related distress and lack of provider recommendation. Healthcare providers generally supported HPV vaccination; however, recommendations were sometimes influenced by perceived sexual activity, age or consent. School-based vaccination was considered useful but required tailored information, preparation, individual adjustments and follow-up. Conclusions: Evidence on HPV vaccination-related knowledge, attitudes and perceptions among adolescents with intellectual disabilities and their stakeholders remains limited and geographically restricted. Improving equitable access may require interventions that should be further investigated, such as accessible communication, systematic provider recommendation, flexible vaccination pathways and greater involvement of adolescents in decision-making.

## 1. Introduction

### 1.1. Rationale

Human papillomavirus (HPV) is one of the most common sexually transmitted infections worldwide. Persistent infection with oncogenic HPV types is a necessary cause of cervical cancer and accounts for a substantial proportion of other anogenital cancers (vulvar, vaginal, penile and anal) and oropharyngeal cancers [1,2]. Globally, HPV-attributable cancers account for an estimated 4.5% of all cancers, corresponding to approximately 630,000 new cancer cases per year, with cervical cancer alone accounting for 83% of this burden [1]. In 2020, the World Health Organization (WHO) launched a global strategy to accelerate the elimination of cervical cancer as a public health problem, setting the “90-70-90” targets to be reached by 2030: vaccinating 90% of girls against HPV by age 15, screening 70% of women with a high-performance test, and treating 90% of women with cervical disease [2,3]. HPV vaccination programs have consistently been shown to be safe and highly effective, with real-world data documenting significant reductions in genital HPV infection and HPV-related disease following their introduction [4]. The number of countries introducing HPV vaccination has continued to expand, from 107 of the 194 WHO Member States (55%) in 2020 to 148 (76%) by early 2025, and the recent adoption of simplified, WHO-endorsed single-dose schedules is expected to further ease program implementation and delivery [5,6]. Nevertheless, global coverage remains far below the 2030 target: population-weighted coverage for the final HPV dose was estimated at only 15% in 2019 and had reached just 20% by 2023, with only a small minority of countries achieving the 90% coverage target [6,7]. Achieving equitable HPV vaccination therefore requires that programs reach all adolescents, including population subgroups that are more likely to be overlooked by routine immunization services.

Lack of confidence in vaccines has increasingly been recognized as a threat to the success of immunization programs, shaped by individual, socio-cultural and trust-related factors rather than by safety and efficacy data alone [8]. HPV vaccine uptake in particular is influenced by hesitancy-related determinants common to vaccination in general, including concerns about vaccine safety, low perceived need, and lack of a clear recommendation from a trusted healthcare provider [9,10]. These general mechanisms of hesitancy may be compounded by additional, population-specific barriers among adolescents with intellectual disabilities and their families.

Adolescents with intellectual disabilities and other neurodevelopmental conditions already experience well-documented inequities in access to healthcare, including preventive and sexual and reproductive health services, despite having rates of sexual activity comparable to their peers without disabilities and a higher risk of non-consensual sexual experiences [11]. Misconceptions about the sexual activity of people with intellectual disabilities nonetheless remain widespread among parents, caregivers and, at times, healthcare providers [12], and may translate into an underestimation of the need for prevention of STIs (e.g., HPV vaccination) in this group [13,14].

Indeed, growing evidence suggests that adolescents with intellectual disabilities and other neurodevelopmental or mental health conditions have lower HPV vaccination coverage than the general adolescent population. A recent commentary highlighted that HPV vaccine uptake is lower among girls with a diagnosis of a mental health condition, and lowest among those with autism or intellectual disability, findings consistent across cohorts from different countries [15]. In line with this, a scoping review of HPV vaccination coverage and determinants in adolescent girls with disability found coverage estimates ranging from 22.9% to 87.4% across studies, with more than half of the studies that included a comparison group reporting lower coverage among girls with disability than among their peers without disability [11].

Despite this emerging evidence on coverage disparities, less is known about the underlying knowledge, attitudes and perceptions regarding HPV vaccination among adolescents with intellectual disabilities themselves, as well as among the parents, caregivers, educators, healthcare workers and other stakeholders involved in vaccination-related decisions on their behalf. Existing reviews on this topic have addressed related but distinct questions: a scoping review on HPV vaccination in mental health populations focused on acceptability, access and uptake across a broad range of mental health conditions [16], while the scoping review by Kumar and colleagues focused specifically on coverage rates and their socio-ecological determinants among adolescent girls with disability [11]. To our knowledge, no systematic review has specifically synthesized the available evidence on knowledge, attitudes and perceptions regarding HPV vaccination for adolescents with intellectual disabilities, integrating the perspectives of adolescents themselves alongside those of their parents/caregivers, educators and healthcare workers.

### 1.2. Objectives

Understanding these perspectives is essential to design HPV vaccination strategies that are accessible, acceptable and tailored to the needs of this population. Therefore, the present systematic review aims to describe the knowledge, attitudes and perceptions regarding HPV vaccination for adolescents with intellectual disabilities, as reported by adolescents themselves, their parents/caregivers, educators, healthcare workers and any other relevant stakeholders.

## 2. Materials and Methods

This review was conducted according to the Preferred Reporting Items for Systematic Reviews and Meta-Analyses (PRISMA) guidelines [17] and according to a pre-specified protocol registered in PROSPERO (CRD420251015183). The review question was: What are the knowledge, attitudes, and perceptions of adolescents with intellectual disabilities, their parents/caregivers, educators, healthcare workers, and other relevant stakeholders regarding HPV vaccination for adolescents with intellectual disabilities?

The review was structured according to the following PICO framework: the population of interest comprised adolescents with intellectual disabilities and key stakeholders, including parents/caregivers, educators, healthcare workers, and other relevant figures; the intervention/exposure was HPV vaccination; no comparator was applicable; and the outcomes of interest were knowledge, attitudes, and perceptions regarding HPV vaccination.

### 2.1. Eligibility Criteria

We included studies that reported original data on knowledge, attitudes, or perceptions regarding HPV vaccination for adolescents with intellectual disabilities. Eligible populations included adolescents with intellectual disabilities and/or key stakeholders involved in their vaccination-related decision-making or care, including parents or caregivers, educators, healthcare workers, school staff, immunization providers, and other relevant figures.

Quantitative, qualitative, and mixed-methods studies were eligible for inclusion, regardless of country or setting. Peer-reviewed journal articles were considered eligible. Studies were included if sufficient information was available to assess their relevance to the review question.

We excluded studies that did not specifically address adolescents with intellectual disabilities or did not provide data relevant to this population or its stakeholders. Studies focused solely on HPV epidemiology, vaccine efficacy/effectiveness, vaccine coverage, or vaccination uptake without reporting knowledge, attitudes, perceptions, barriers, facilitators, or reasons for vaccination acceptance, hesitancy, or refusal were excluded. Studies assessing the general population without a clear subgroup or stakeholder perspective relevant to adolescents with intellectual disabilities were also excluded. Records were excluded if they did not report original data relevant to the review question, including editorials, commentaries, opinion pieces, and narrative articles without primary data. Case reports were also excluded. Studies not available in full-text format were excluded; however, conference abstracts were considered eligible when they reported original data and provided sufficient information to assess relevance to the review question.

### 2.2. Information Sources and Search Strategy

A systematic search was conducted in PubMed, Embase, and Scopus using a pre-defined search strategy developed by the review team (Supplementary Material S1. Search Strategy). The search strategy combined terms related to intellectual disability, developmental disability, adolescents, HPV, and HPV vaccination. No date and language restrictions were applied.

The search was conducted on 29 October 2025. Reference lists of all included studies were screened to identify additional eligible records. In addition, any systematic reviews, scoping reviews, or other review articles retrieved during the search process were not included as primary studies, but their reference lists were checked to identify further potentially eligible original studies.

### 2.3. Selection Process

Search results were imported into Rayyan [18], and duplicates were removed before screening. Titles and abstracts were screened independently by at least two reviewers. Full texts were then assessed independently by at least two reviewers against the pre-specified eligibility criteria. At both screening stages, reviewers were blinded to each other’s decisions. Disagreements were resolved through consensus discussion; when necessary, a third reviewer was involved.

### 2.4. Data Collection Process and Data Items

Data were extracted using a standardized Excel spreadsheet developed for this review. For each included study, one reviewer extracted the data and at least one second reviewer checked the extraction for accuracy and completeness. Disagreements were resolved through consensus discussion.

The following information was extracted: author, year of publication, country, setting, year of study, study design, participant characteristics, sample size, age and gender when reported, relevant measures or data collection methods, and findings related to knowledge, attitudes, and perceptions of HPV vaccination, reasons for HPV vaccine refusal when these were reported by participants or stakeholders, funding, and conflict of interest.

### 2.5. Study Risk of Bias Assessment

The methodological quality of included studies was assessed using the Joanna Briggs Institute Critical Appraisal Checklists. Qualitative studies were assessed using the JBI Critical Appraisal Checklist for Qualitative Research [19]. Cross-sectional quantitative studies were assessed using the JBI Critical Appraisal Checklist for Cross-sectional Studies (Prevalence studies) [20]. Each included study was assessed independently by at least two reviewers. Disagreements were resolved through consensus discussion. The risk of bias assessment was used to inform the interpretation of findings but was not used as an automatic criterion for exclusion.

### 2.6. Effect Measures, Reporting Bias Assessment, and Certainty

Because of the narrative synthesis and the heterogeneity of study designs, populations, stakeholder groups, data collection methods and outcomes, no common effect measure was defined or calculated. Quantitative findings were reported using the measures provided in the original studies, including counts, percentages and item-level responses where available. Formal assessment of reporting bias, including publication bias, was not performed because only a small number of heterogeneous studies were included, and no meta-analysis was conducted. No formal certainty of evidence assessment was undertaken, as the evidence consisted of diverse qualitative and descriptive quantitative studies without a single intervention effect or comparable outcome.

### 2.7. Synthesis Methods

A narrative synthesis was conducted. Findings were organized by stakeholder group (adolescents with disabilities, parents/caregivers, healthcare providers, and school staff). Quantitative findings were described using the measures reported in the original studies, including counts, percentages and item-level responses where available. Qualitative findings were synthesized narratively by identifying recurring themes across studies. No meta-analysis was conducted because of heterogeneity in study design, populations, data collection methods and outcome measures.

## 3. Results

### 3.1. Study Selection

**Figure 1:**
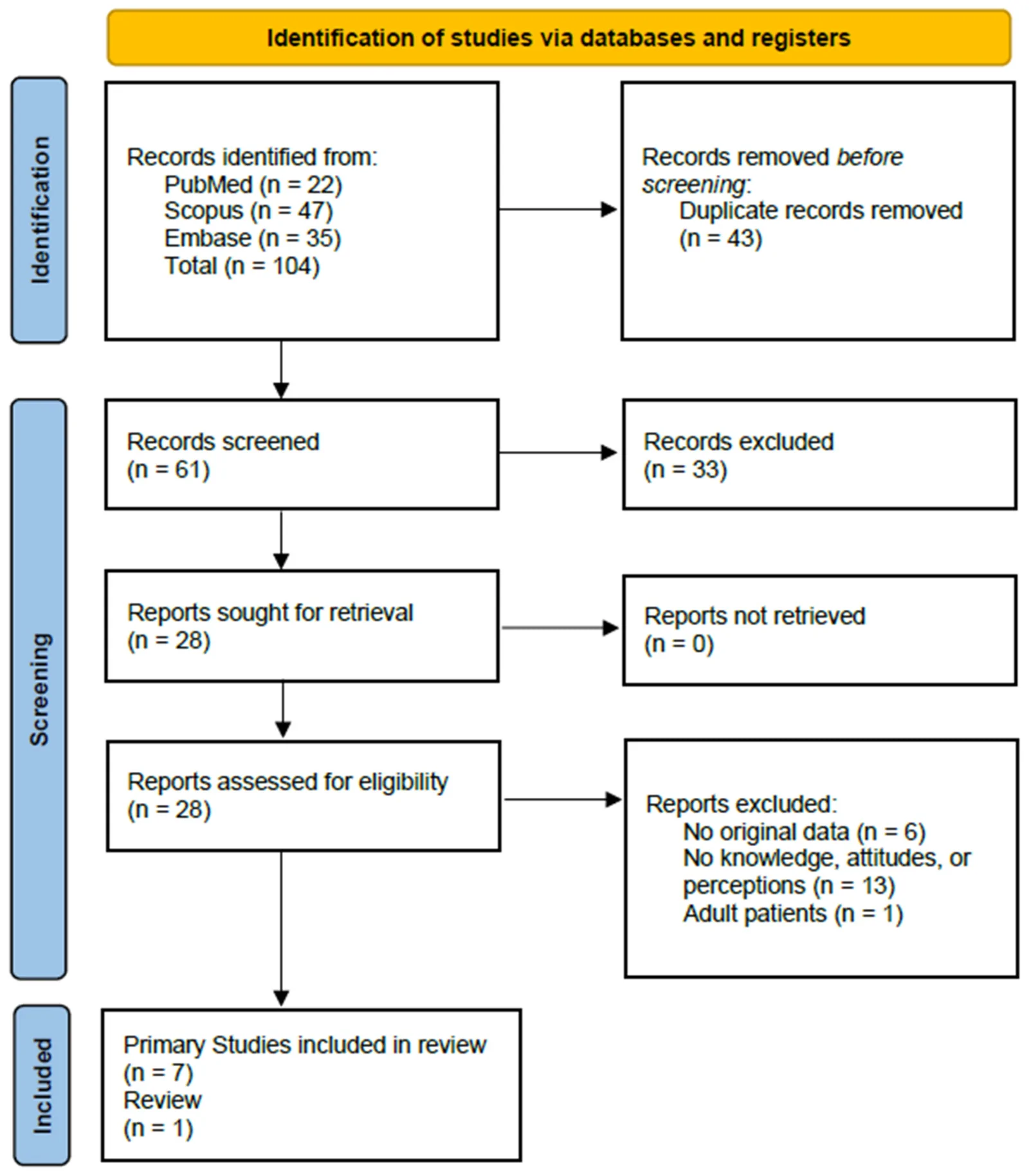
Flow diagram.

The database search identified 104 records (Figure 1). After duplicate removal, 61 unique records were screened by title and abstract. Overall, 28 records were selected for full-text assessment. At this stage, seven primary studies met the inclusion criteria and were included in the review, while one scoping review [16] was retained only for reference list checking. Screening the reference list of the scoping review did not identify any additional eligible studies. Reference list checking of the eight included studies identified the full-length main paper [21] corresponding to one conference abstract initially included among the primary studies [22]; the full paper [21] was therefore considered in the present synthesis.

### 3.2. Study Characteristics

The seven included studies were published between 2013 [21] and 2025 [23] and were conducted in the USA (*n* = 4) [21,24,25,26], Australia (*n* = 2) [27,28], and the UK (*n* = 1) [23]. Only one study was published before 2021 [21]. When reported, data collection took place between 2009–2010 [21] and 2022 [28]. All studies were published in English.

Settings included clinical services or specialist clinics (*n* = 3) [21,25,26], educational institutions or special schools (*n* = 3) [23,27,28], and online surveys (*n* = 2) [21,24]. Study designs comprised quantitative cross-sectional surveys (*n* = 2) [21,25], one retrospective chart review with a prospective caregiver survey [26], and qualitative studies (*n* = 4) [23,24,27,28]. Quantitative studies used online or parent-completed questionnaires [21,25], while Humphrey combined medical record review with a caregiver survey [26]. Qualitative studies used online open-ended surveys [24], activity-oriented focus groups with visual aids and accessible materials [23], focus groups and semi-structured interviews [27], or online semi-structured qualitative interviews [28].

Participants included parents/caregivers (*n* = 5) [21,25,26,27,28], physicians or other healthcare providers (*n* = 4) [21,24,27,28], school staff (*n* = 2) [27,28], and adolescents with disabilities (*n* = 2) [23,28]. Target populations included adolescents or young people with intellectual disability or broader special healthcare needs including intellectual disability [21,23,24,27,28], with autism spectrum disorder [25,27,28], and with Rett syndrome [26]. Four studies focused exclusively on female target populations [21,24,25,26], one exclusively on young men [23], and two included participants regardless of sex/gender [27,28] (Table 1).

All studies reported conflict-of-interest information, while all except one [21] reported funding information.

### 3.3. Results of Individual Studies

#### 3.3.1. Adolescents with Disabilities

Evidence directly collected from adolescents or young people with disabilities was available in two qualitative studies [23,28].

In Carnegie et al., young men with mild to moderate intellectual disabilities had limited understanding of HPV and described exclusion from HPV-related information and school vaccine discussions. The study also highlighted communication barriers in conveying abstract concepts such as HPV infection, cancer prevention and future sexual health risk, suggesting the need for activity-based communication, concrete artefacts, visual or sensory materials, easy-read resources, videos, face-to-face sexual health education, and sufficient time to discuss both factual and emotional aspects of HPV vaccination [23]. In Tuckerman et al., adolescents with intellectual disability or autism showed limited awareness of vaccine benefits and tended to associate vaccination with pain, discomfort or anxiety [28].

Overall, the limited evidence from adolescents themselves suggests that HPV vaccination was often poorly understood because information was not sufficiently accessible, inclusive or adapted to their communication needs.

#### 3.3.2. Parents and Caregivers

Parent and caregiver data were reported in five studies [21,25,26,27,28]. Overall, parents were often described as broadly supportive of vaccination, but HPV-specific knowledge, perceived need, and concerns varied across studies.

In Cody, HPV knowledge was assessed through seven items. Most parents correctly identified HPV as a sexually transmitted infection, a cause of genital warts and a possible cause of abnormal Pap smear findings; however, more than one-third scored below 57% on the knowledge items. General vaccine acceptance was relatively high: 12/17 parents accepted all vaccines, while 4/17 had refused at least one vaccine. HPV vaccine offer and uptake were lower: 8/17 reported that their daughter’s pediatrician had offered HPV vaccination, and 3/17 reported that their daughter had received it [21].

Perceived low susceptibility to HPV was reported across several studies. In Cody, 5/17 parents agreed that their child was unlikely to be infected with HPV, and 4/17 agreed that children with disabilities are less likely to acquire HPV [21]. In Carter, some parents questioned the need for HPV vaccination because they did not expect their child to become sexually active [27]. In Humphrey, among HPV vaccine-eligible participants with available vaccination data, 11/48 (22.9%) had received HPV vaccination according to recommendations. Among those not vaccinated, the most common guardian-reported reasons for HPV non-vaccination were that the vaccine was considered unnecessary (11/35; 31.4%) and that the patient was not sexually active (8/35; 22.9%) [26].

Safety and procedure-related concerns were also reported. In Chen, the most frequent reason for HPV vaccine non-receipt among unvaccinated children was concern about side effects (*n* = 9), followed by lack of recommendation or offer by a provider (*n* = 7), perception that the child was too young (*n* = 7), other reasons (*n* = 6) and inconvenience (*n* = 3) [25]. In Humphrey, additional reasons included concerns about unknown long-term effects (7/35; 20.0%), competing complex health needs (5/35; 14.3%), absence of physician recommendation (3/35; 8.6%) and cost (1/35; 2.9%) [26]. In Carter, parents’ concerns included adverse effects, beliefs linking vaccines to autism, COVID-19-related vaccination fatigue, needle anxiety and possible procedure-related trauma [27].

Tuckerman reported high parental trust in vaccines, government and healthcare providers, with no clear difference in attitudes toward HPV vaccination compared with other routine vaccines. However, parents lacked accessible information and wanted clearer information about vaccination options, additional support and vaccination pathways outside the school program [28].

#### 3.3.3. Healthcare Providers and Immunization Staff

Healthcare provider or immunization staff perspectives were reported in four studies [21,24,27,28]. Across studies, professionals generally supported HPV vaccination, but reported barriers related to parental concerns, consent, communication and service delivery.

In Bouza, all physicians rated HPV vaccination as important for females with intellectual disability: 34/51 rated it “very important” and 17/51 “important”. Most had recommended HPV vaccination in practice (49/51), and 37/51 felt an obligation to try to convince patients or caregivers to consent. The main reasons given for vaccine importance were prevention of HPV and cancer (45.1%), the same reasons as for other patients (22.5%), and high risk of sexual abuse (12.7%) [24].

Provider decision-making sometimes included sexual activity and consent considerations. In Bouza, the most frequent factors considered before recommending HPV vaccination were sexual activity (24.8%), age (22.0%) and ability to obtain informed consent (11.4%) [24]. In Cody, providers perceived parental reluctance as a major barrier: 51/77 considered parents likely to be reluctant to vaccinate against a sexually transmitted infection, and 39/77 considered parents likely to be reluctant to discuss sexuality or sexually transmitted infections [21].

Provider-level barriers were less frequent than parent-level barriers. Among providers who saw children with special healthcare needs and offered vaccination, 69/77 had vaccinated patients with disabilities against HPV. The main reported reason for not administering HPV vaccination was parental refusal (6/77; 7.8%). Other reported barriers included difficulty getting older children or adolescents to attend well visits or immunization visits (28/77; 36%) and severe disability requiring assistance with all activities of daily living (21/77; 27%). Only 4/77 identified personal reluctance to discuss sexuality or sexually transmitted infections as a likely barrier [21].

Service and communication barriers were also highlighted. In Carter, staff described standard vaccination information as insufficiently tailored for families of students with disability, particularly in the presence of low health literacy or high support needs. Limited direct communication between parents and immunization providers was also reported [27]. In Tuckerman, barriers included inaccessible information, information overload, paper consent processes, limited routes for parent questions, attendance issues, vaccine-related anxiety, behavioral challenges and limited follow-up after absence or unsuccessful vaccination. Reported facilitators included school-based delivery, preparation before vaccination day, school–council collaboration, individual tailoring and additional support outside school [28].

#### 3.3.4. School Staff and Educational Settings

School staff perspectives were reported in two studies [27,28]. Across both studies, school-based vaccination was described as potentially convenient and familiar, but dependent on accessible information, preparation, individual adjustments and coordination between schools and immunization services.

In Carter, school staff considered the standard vaccination information pack inadequate for some families and reported that parents often needed more tailored information. Staff also described limited direct communication between parents and immunization providers [27].

In Tuckerman, specialist schools were described as supportive vaccination settings when students were prepared in advance, and vaccination was adapted to individual needs. Barriers included consent processes, limited parent information routes, attendance, facilities or space, anxiety, behavioral challenges and limited follow-up after missed or unsuccessful vaccination [28].

Table 2 summarizes knowledge, attitudes, and perceptions across the included studies.

### 3.4. Risk of Bias in Studies

Full risk of bias evaluation is reported in Supplementary Material S2. Risk of bias.

Overall, the four qualitative studies showed good congruity between the research questions, methodology, data collection methods, analysis and interpretation of findings. Participants’ voices were adequately represented, and the conclusions were supported by the reported results in all four studies. Ethical approval was reported in all studies. The main methodological limitations concerned incomplete reporting of the underlying philosophical perspective and limited reflexivity: most studies did not clearly describe the researchers’ positionality or the potential influence of the researchers on data collection and interpretation.

Considering the three quantitative studies, sample frames were broadly appropriate to the disability-specific populations addressed, but limitations concerned unclear or convenience-based sampling and small sample sizes. Outcomes were generally measured consistently within studies; however, reliance on self- or proxy-reported data may have introduced measurement bias. Analyses were mainly descriptive and appropriate for the study aims, but confidence intervals were not always reported. The main concerns related to incomplete reporting or low response rates, particularly for provider surveys and caregiver follow-up components.

### 3.5. Results of Syntheses, Reporting Biases, and Certainty of Evidence

No common effect measure was calculated, and no meta-analysis was conducted because of heterogeneity in study design, populations, stakeholder groups and outcomes. Formal assessment of reporting bias and certainty of evidence was not performed because the review included a small number of heterogeneous qualitative and descriptive quantitative studies without pooled estimates or comparable intervention effects.

## 4. Discussion

The available data from the scientific literature show that HPV vaccination coverage in adolescents with intellectual disabilities may be lower compared with the general population [11,15]. In order to better understand this issue, the present review aimed to address the knowledge, attitudes and perceptions of adolescents with intellectual disabilities, their parents/caregivers, educators, healthcare workers and any other relevant stakeholders regarding HPV vaccination for adolescents with intellectual disabilities.

Overall, the results from the present work underline that further studies are needed to fully understand this crucial issue and to ensure equitable access to HPV vaccination campaigns. Most of the studies included in the review have been published in recent years [23,24,25,26,27,28], highlighting the growing attention of the scientific community on the topic. Nonetheless, only seven studies are available, with small sample sizes and varying sample composition. Moreover, all studies were conducted in Western countries, namely the USA, Australia and the UK, limiting the transferability of findings to healthcare systems, school vaccination models and sociocultural contexts outside these settings. Therefore, the findings should not be interpreted as globally generalizable to all adolescents with intellectual disabilities, particularly in low- and middle-income countries, non-English-speaking contexts, or healthcare systems with different vaccination delivery models. Only two studies directly collected the perspectives of adolescents receiving the preventive intervention [23,28], showing that the voices of adolescents with disabilities themselves remain poorly represented in the available literature.

Our findings suggest that some determinants of HPV vaccine hesitancy identified in this population overlap with those described in the general population, such as fear of side effects, the perception that the vaccine is not necessary, or lack of a physician recommendation [9]. However, most included studies did not include comparison groups from the general adolescent population; therefore, this review cannot quantify the excess burden of barriers among adolescents with intellectual disabilities. Potentially population-specific barriers were difficulties in accessing information [23,28], caregiver dependence, communication difficulties, procedure-related anxiety, and organizational problems during vaccination [21,25,26,27]. The interpretation of these additional barriers is based on the comparison between themes identified in the included studies and determinants previously described in the general population and should therefore be interpreted as potential additional barriers rather than as directly measured differences.

A central theme emerging from the review was the lack of accessible and tailored communication. From the point of view of adolescents, the available evidence suggests limited understanding of HPV and HPV vaccination, together with exclusion from school vaccine discussions and sexual health information [23,28]. Standard written or verbal communication may be insufficient to explain abstract concepts such as HPV infection, cancer prevention and future sexual health risk. This indicates the need for accessible, developmentally appropriate and multimodal communication strategies.

An important theme related to HPV vaccination attitudes concerned perceptions of the sexual activity of people with intellectual disabilities. Indeed, the review shows that several studies reported that parents, caregivers or healthcare professionals sometimes assumed that adolescents or young people with intellectual disabilities were unlikely to be sexually active, and that this could affect the perceived relevance of HPV vaccination [21,24,26,27,28]. Similar assumptions also appeared to influence some healthcare professionals’ decision-making, as sexual activity was among the factors considered before recommending HPV vaccination in one study [24]. However, the included studies were cross-sectional or qualitative and did not test a causal pathway between assumptions about sexual inactivity, perceived susceptibility and vaccine refusal. Therefore, this pathway should be interpreted as a plausible explanatory mechanism suggested by recurring qualitative and descriptive findings, rather than as a demonstrated causal association. Notably, evidence among adolescents and young people with intellectual disabilities indicates that many may be romantically and sexually active and may experience specific sexual health risks, including unsafe sex, limited sexual knowledge and vulnerability to sexual or dating violence [29,30,31]. Education and health services should therefore avoid basing HPV vaccine recommendations on presumed future sexual inactivity and should operate on the assumption that young people with intellectual disabilities require the same access to sexual and reproductive health prevention as their peers.

Addressing this issue is crucial to overcome stereotypes that may undermine the right of persons with disabilities to access proper sexual and reproductive health information and services. Currently, a limited number of studies have been conducted to deeply understand the needs and to develop relevant interventions for promoting the sexual health of young people with intellectual disabilities [32,33,34,35]. Furthermore, this is even more important considering the “3 Cs” model [10] that identified complacency as one of the three main categories of vaccine hesitancy. In particular, complacency is present where perceived risks of vaccine-preventable diseases are low, and vaccination is not deemed necessary [10]. In this review, perceived low susceptibility to HPV infection, often linked to assumptions about sexual inactivity, appeared to be a relevant form of HPV vaccine complacency in this population.

For these reasons, the role of healthcare professionals becomes crucial to ensure proper communication not only regarding the benefits and the risks of vaccination itself but also regarding the risks of possible HPV infection. The review results highlight that, although healthcare professionals show a good level of acceptance of vaccination, there are some gaps in communication with adolescents and their reference adults [21,24,27]. Some providers perceived parents as the main barrier to HPV vaccination, especially because of concerns about sexuality, vaccination against a sexually transmitted infection, and vaccine safety [21]. At the same time, the findings suggest that healthcare professionals should be supported in framing HPV vaccination as a standard preventive intervention rather than as a decision dependent on presumed sexual activity, disability severity or caregiver expectations.

Communication emerged as one of the central elements, highlighting the need to dedicate the right amount of time to counseling, which must include adolescents with intellectual disabilities in the decision process, providing them with specific materials and giving their caregivers simple information. Although not specific to HPV vaccination or sexual health services, broader evidence from pediatric disability services indicates that parents often value individualized, coordinated and accessible care; respectful communication; and collaboration with service providers [36].

In this regard, the few available studies included in this review highlight how adolescents with intellectual disabilities are occasionally involved in the decision-making process, which is almost entirely delegated to the caregivers. Nevertheless, the United Nations Convention on the Rights of Persons with Disabilities (UNCRPD) affirms the right for people with disabilities to make decisions and self-determine [37]. In this context, healthcare decision-making by individuals with intellectual disabilities is increasingly recognized across clinical practice, policy, and legal contexts [38]. Supported decision-making promotes a patient-centered approach by enabling individuals to make their own decisions with appropriate support from trusted people, such as family members, friends or professionals [39]. Recent empirical research further highlights that supported decision-making depends on relational support, accessible communication, attention to the person’s will and preferences, and careful involvement of family members or other supporters [40,41,42]. At the moment, few studies are examining interventions for healthcare decision-making for children with intellectual disability [39].

Considering the distinctive features of this specific group, the analyzed literature underscored the perceived usefulness of school-based vaccination programs, tackling some of the challenges in accessing HPV vaccinations [27,28]. These results are consistent with previous studies performed in the general population, which outlined that school-based interventions and HPV vaccination programs in school are effective not only to improve vaccination rates but also to increase knowledge and awareness of the risk of HPV infection and the importance of HPV vaccination [43,44,45]. Nevertheless, few studies have been performed to fully understand how to achieve the maximum effectiveness of school-based programs, which have to be tailored taking into account the specific needs of minors with intellectual disabilities and their families [46]. School-based programs for adolescents with intellectual disabilities or autism cannot simply replicate standard models. The included studies highlighted that school staff and immunization providers perceived a need for tailored information, preparation before vaccination day, individual adjustments, accessible consent processes, clear communication with parents, and follow-up after missed or unsuccessful vaccination attempts [27,28].

The interpretation of these findings should also consider the heterogeneity of the populations included in the review. Intellectual disability is a broad category that includes individuals with very different cognitive, communicative and support needs. Moreover, some included studies involved broader or related populations, such as adolescents with autism spectrum disorder, special healthcare needs, or Rett syndrome. Although these groups may share barriers related to accessibility, communication and support needs, their experiences cannot be assumed to be interchangeable. Subgroup analyses by disability type or severity were not performed. Given the small number of included studies, overlapping diagnostic groups, heterogeneous samples and limited reporting of disability severity, stratifying the findings would have risked producing artificial distinctions or unsupported subgroup conclusions. The findings should therefore be interpreted as describing recurring issues across a heterogeneous evidence base, rather than as recommendations that apply uniformly to all disability subgroups. This heterogeneity limits the possibility of drawing conclusions that apply equally to all adolescents with intellectual disabilities and underlines the need for future studies with clearer definitions of disability groups and more detailed reporting of participants’ characteristics.

This review had several limitations. Only three databases were searched, and gray literature was not included. The number of included studies was small, and the available evidence was geographically limited. Study designs, populations, data collection methods and outcome measures were heterogeneous, preventing meta-analysis and limiting comparability across studies. Several studies had small samples, and some relied on self- or proxy-reported data. In addition, only two studies directly included adolescents or young people with disabilities. Thus, the limited direct involvement of adolescents is an important limitation of the evidence base. Most findings were derived from parents, caregivers, healthcare professionals or school staff, whose views may not fully reflect adolescents’ own knowledge, preferences, concerns or decision-making priorities. Proxy perspectives are relevant because these stakeholders often influence vaccination decisions, but they cannot replace direct evidence from adolescents themselves. These aspects should be considered when interpreting the findings. Temporal changes in HPV vaccination policies, gender-neutral recommendations, public awareness and vaccine confidence should also be considered. Because of the small number of studies and heterogeneity of outcomes, a stratified analysis by data collection period was not performed. However, attitudes and barriers reported in earlier studies may not fully reflect current HPV vaccination contexts. Further research is needed to improve the quality and comparability of evidence in this field. Future studies should include larger and more diverse samples, use accessible methods to directly involve adolescents with intellectual disabilities, and apply clearer definitions of disability groups. Standardized measures of HPV-related knowledge, attitudes and perceptions would improve comparability across studies.

Although the included studies did not provide interventional evidence, adapted communication strategies and flexible vaccination pathways emerged from reported barriers, facilitators and stakeholder suggestions. These approaches should therefore be interpreted as candidate strategies for future intervention development and evaluation. Future interventional studies are needed to assess whether they improve HPV-related knowledge, acceptability and vaccination uptake in this population.

In conclusion, given what has emerged from the present review, the standard approach may not be sufficient to ensure equitable access to vaccination for adolescents with intellectual disabilities, requiring adaptation through flexible vaccination pathways, personalized counseling, greater collaboration between vaccination services and the child’s living environment, and the involvement of parents. To achieve these goals, education and training of the involved healthcare workers and school staff are crucial.

## 5. Conclusions

Evidence on HPV vaccination-related knowledge, attitudes and perceptions among adolescents with intellectual disabilities and their stakeholders remains limited and geographically restricted. Improving equitable access may require interventions that should be further investigated, such as accessible communication, systematic provider recommendation, flexible vaccination pathways and greater involvement of adolescents in decision-making.

## Figures and Tables

**Table 1:** Characteristics of studies.

First Author Year	Country	Setting Year of Study	Study Design	Participants	Target Group
Bouza B 2021 [24]	USA	Online Year: N.A.	Cross-sectional open-ended survey with inductive content analysis	51 physicians in pediatrics, family medicine or obstetrics and gynecology with prior experience caring for female patients with intellectual disability Participants mean age 46.6 years (SD = 11.5), range 29–72	Female patients with intellectual disability
Carnegie E 2025 [23]	UK	Education institutions 2015–2016	Qualitative focus group study using critical discourse analysis	18 young men with mild to moderate intellectual disabilities attending further education institutions; able to consent and engage in short-sentence conversation Participants age: 16–22 years	Young men with mild to moderate intellectual disability
Carter A 2024 [27]	Australia	Special schools Year: N.A.	Qualitative study with thematic analysis	40 adult stakeholders involved in vaccination for students with intellectual disability and/or autism attending special schools 6 mothers, 24 education staff/school stakeholders, 10 health/immunization staff Mothers age: 40–52 years. Other: N.A.	Students with intellectual disability and/or autism attending special schools
Chen Y 2024 [25]	USA	Tertiary neurodevelopmental disorders clinic Year: N.A.	Cross-sectional study	73 parents/guardians of females with autism spectrum disorder aged 11–17 Participants age: N.A.	Females with autism spectrum disorder; approximately 30 participants also had intellectual disability and/or language impairment.
Cody PJ 2013 [21]	USA	Parents: outpatient urban health clinic and Children’s Hospital of Adolescent Medicine and Neurology clinics Providers: online 2009–2010	Cross-sectional study	17 parents of female children with special healthcare needs (CSHCN) aged 9–26 years 77 provider respondents seeing CSHCN and offering vaccination as part of routine practice Parents age: 33–54 years (CSHCN age 12–22 years, mean 15.6) Providers age: N.A.	Female children with special healthcare needs across disability categories, including autism, developmental delay, intellectual disability, multiple disabilities and other categories; broader than intellectual disability only
Humphrey KN 2021 [26]	USA	Rett syndrome specialty clinic 2013–2018	Retrospective cross-sectional chart review plus prospective caregiver survey	77 female patients with Rett syndrome Reasons for non-vaccination available for 35 caregivers Patients age: 12–55 years Caregivers age: N.A.	Female patients with classic or atypical Rett syndrome
Tuckerman J 2024 [28]	Australia	Special schools 2022	Qualitative interview study; themes were inductively generated through thematic analysis and then deductively mapped to the UNICEF “Journey to Immunization” model	32 stakeholders associated with specialist schools for intellectual disability or autism 2 adolescents (1 male), 7 parents (1 male), 13 school staff, 10 council immunization staff Participants age: N.A.	Adolescents with intellectual disability or autism in specialist schools

Abbreviations: CSHCN, children with special healthcare needs; N.A., not available; SD, standard deviation; UK, United Kingdom; UNICEF, United Nations Children’s Fund; USA, United States of America.

**Table 2:** Knowledge, attitudes, and perceptions regarding HPV vaccination across included studies.

First Author Year	Participants	Knowledge	Attitudes/Perceptions and Barriers/Facilitators
Bouza B 2021 [24]	Physicians	N.A.	Physicians considered HPV vaccination important/very important for females with ID (51/51). Main reasons: HPV/cancer prevention, same indications as other patients, sexual abuse risk. Recommendation influenced by sexual activity, age and consent. Caregivers described as mostly receptive but often concerned about side effects, vaccine need, guidelines and perceived sexual inactivity.
Carnegie E 2025 [23]	Young men with mild to moderate intellectual disabilities	Limited or no HPV understanding; variable cancer understanding; exclusion from HPV/school vaccine information	Participants described exclusion from public health information and sexual-health discourse. Barriers: inaccessible HPV information, overprotection, HPV framed as private, difficulty understanding abstract HPV concepts. Facilitators: visual/activity-based resources, concrete materials, easy-read information, face-to-face education.
Carter A 2024 [27]	Mothers, education staff/school stakeholders, health/immunization staff	Misconceptions about HPV, autism, boys’ vaccination and sexual inactivity	Parents described as mostly pro-vaccination, but some hesitant/anti-vaccination. Barriers: safety concerns, autism-related misconceptions, COVID-19 vaccine fatigue, perceived sexual inactivity, needle/procedure-related distress, limited tailored information and parent-provider communication. Staff reported need for accessible, personalized communication.
Chen Y 2024 [25]	Parents/guardians	N.A.	Reasons for HPV non-receipt among unvaccinated children: side effects, no provider recommendation/offer, child considered too young, other reasons, inconvenience.
Cody PJ 2013 [21]	Parents and providers	Most parents knew HPV is an STI and related to genital warts/abnormal Pap smears, but >1/3 scored < 57%.	Parents: general vaccine acceptance high, but HPV offer/uptake low; mixed HPV-specific perceived benefit and low perceived susceptibility. Providers: high HPV vaccine acceptability; 69/77 vaccinated patients with disabilities. Main barriers: parental refusal, reluctance to vaccinate against an STI, reluctance to discuss sexuality/STIs, missed adolescent visits, severe disability.
Humphrey KN 2021 [26]	Caregivers	N.A.	Reasons for non-vaccination: vaccine considered unnecessary, patient not sexually active, unknown long-term effects, competing complex health needs, no physician recommendation, cost.
Tuckerman J 2024 [28]	Adolescents, parents school staff, council immunization staff	Parents had high trust in vaccines but limited HPV knowledge. Adolescents had limited awareness of benefits and associated vaccination with pain/anxiety.	Most parents supported vaccination; no clear difference between HPV and other routine vaccines. Barriers: inaccessible information, information overload, low health literacy/CALD needs, limited understanding of HPV relevance for boys/disability, paper consent, limited parent questions, anxiety/behavioral challenges, attendance/follow-up issues. Facilitators: school-based delivery, preparation, individual tailoring, school–council collaboration, additional support outside school.

Abbreviations: CALD, culturally and linguistically diverse; ID, intellectual disability; N.A., not available; STI, sexually transmitted infection.

## Data Availability

No new data were created or analyzed in this study. Data sharing is not applicable to this article.

## Supplementary Materials

The following supporting information can be downloaded at: https://www.mdpi.com/article/10.3390/vaccines14090764/s1, Supplementary Material S1: Search strategy; Supplementary Material S2: Risk of bias.

## Abbreviations

The following abbreviations are used in this manuscript:

CALD	Culturally and linguistically diverse
CSHCN	Children with special healthcare needs
HPV	Human papillomavirus
ID	Intellectual disability
JBI	Joanna Briggs Institute
N.A.	Not available
PICO	Population, Intervention/Exposure, Comparator, Outcome
PRISMA	Preferred Reporting Items for Systematic Reviews and Meta-Analyses
PROSPERO	International Prospective Register of Systematic Reviews
SD	Standard deviation
STI	Sexually transmitted infection
UK	United Kingdom
UNCRPD	United Nations Convention on the Rights of Persons with Disabilities
UNICEF	United Nations Children’s Fund
USA	United States of America
WHO	World Health Organization

## References

[1] de Martel C., Plummer M., Vignat J., Franceschi S. (2017). Worldwide Burden of Cancer Attributable to HPV by Site, Country and HPV Type. Int. J. Cancer.

[2] Malagón T., Franco E.L., Tejada R., Vaccarella S. (2024). Epidemiology of HPV-Associated Cancers Past, Present and Future: Towards Prevention and Elimination. Nat. Rev. Clin. Oncol..

[3] World Health Organization (2020). Global Strategy to Accelerate the Elimination of Cervical Cancer as a Public Health Problem.

[4] Wang W., Kothari S., Skufca J., Giuliano A.R., Sundström K., Nygård M., Koro C., Baay M., Verstraeten T., Luxembourg A. (2022). Real-World Impact and Effectiveness of the Quadrivalent HPV Vaccine: An Updated Systematic Literature Review. Expert Rev. Vaccines.

[5] Bruni L., Saura-Lázaro A., Montoliu A., Brotons M., Alemany L., Diallo M.S., Afsar O.Z., LaMontagne D.S., Mosina L., Contreras M. (2021). HPV Vaccination Introduction Worldwide and WHO and UNICEF Estimates of National HPV Immunization Coverage 2010–2019. Prev. Med..

[6] Kreimer A.R., Porras C., Liu D., Hildesheim A., Carvajal L.J., Ocampo R., Romero B., Gail M.H., Cortes B., Sierra M.S. (2025). Noninferiority of One HPV Vaccine Dose to Two Doses. N. Engl. J. Med..

[7] Han J., Zhang L., Chen Y., Zhang Y., Wang L., Cai R., Li M., Dai Y., Dang L., Chen H. (2025). Global HPV Vaccination Programs and Coverage Rates: A Systematic Review. eClinicalMedicine.

[8] Dubé E., Laberge C., Guay M., Bramadat P., Roy R., Bettinger J.A. (2013). Vaccine Hesitancy. Hum. Vaccines Immunother..

[9] Karafillakis E., Simas C., Jarrett C., Verger P., Peretti-Watel P., Dib F., De Angelis S., Takacs J., Ali K.A., Pastore Celentano L. (2019). HPV Vaccination in a Context of Public Mistrust and Uncertainty: A Systematic Literature Review of Determinants of HPV Vaccine Hesitancy in Europe. Hum. Vaccines Immunother..

[10] MacDonald N.E. (2015). Vaccine Hesitancy: Definition, Scope and Determinants. Vaccine.

[11] Kumar P., Bateson D., Costa T., Ahmed S., Naidu C. (2025). HPV Vaccination Coverage and Determinants in Adolescent Girls with Disability: A Scoping Review. Vaccine.

[12] Pebdani R.N., Tashjian A. (2022). An Analysis of the Attitudes of the General Public Towards the Sexuality of Individuals with Disabilities Through a Systematic Literature Review. Sex. Disabil..

[13] McLean K.J., Sadowsky M., Chesnokova A., Chvasta K., Lee W.-L., Ventimiglia J., Shea L. (2025). Assessing STI and HIV Risks among Autistic Individuals: Implications for Healthcare Access and Intervention. Disabil. Health J..

[14] Schmidt E.K., Brown C., Darragh A. (2020). Scoping Review of Sexual Health Education Interventions for Adolescents and Young Adults with Intellectual or Developmental Disabilities. Sex. Disabil..

[15] Ellingson M.K., Brewer N.T. (2024). Mental Health and Lower Adolescent HPV Vaccine Coverage. Lancet Public Health.

[16] King K.D., Fernandez-Sanchez H., MacDonald S.E. (2024). Acceptability, Access, and Uptake of Human Papillomavirus Vaccination in Mental Health Populations: A Scoping Review. J. Public Health.

[17] Page M.J., McKenzie J.E., Bossuyt P.M., Boutron I., Hoffmann T.C., Mulrow C.D., Shamseer L., Tetzlaff J.M., Akl E.A., Brennan S.E. (2021). The PRISMA 2020 Statement: An Updated Guideline for Reporting Systematic Reviews. BMJ.

[18] Ouzzani M., Hammady H., Fedorowicz Z., Elmagarmid A. (2016). Rayyan—A Web and Mobile App for Systematic Reviews. Syst. Rev..

[19] Porritt K., Evans C., Bennet C., Loveday H., Bjerrum M., Salmond S., Munn Z., Pollock D., Pang D., Vineetha K. (2024). Systematic Reviews of Qualitative Evidence. JBI Manual for Evidence Synthesis.

[20] Munn Z., Moola S., Lisy K., Riitano D., Tufanaru C. (2020). Systematic Reviews of Prevalence and Incidence. JBI Manual for Evidence Synthesis.

[21] Cody P.J., Lerand S.J. (2013). HPV Vaccination in Female Children with Special Health Care Needs. J. Pediatr. Adolesc. Gynecol..

[22] Cody P., Lerand S., Schroeder J., Simpson P., Nugent M. (2010). Protecting Those with Disabilities: HPV Vaccination Practices. J. Adolesc. Health.

[23] Carnegie E., Gray-Brunton C., Kennedy C., Pow J., Willis D., Whittaker A. (2025). Young Men with Intellectual Disabilities’ Perceptions of HPV and HPV Vaccine: A Qualitative Study on How to Communicate HPV Vaccine Information. Hum. Vaccines Immunother..

[24] Bouza B., Hammig B., Schaefer Whitby P. (2021). Physicians’ Experiences of Recommending the HPV Vaccine to Females with an Intellectual Disability. Sex. Disabil..

[25] Chen Y., Powers J., McDougle C.J., Zürcher N.R., Thom R.P. (2024). Cervical Cancer Screening and Prevention Uptake in Females with Autism Spectrum Disorder. J. Autism Dev. Disord..

[26] Humphrey K.N., Horn P.S., Olshavsky L., Reebals L., Standridge S.M. (2021). Well-Woman Care and HPV Vaccination Rates in Women with Rett Syndrome. Disabil. Health J..

[27] Carter A., Klinner C., Young A., Strnadová I., Wong H., Vujovich-Dunn C., Newman C.E., Davies C., Skinner S.R., Danchin M. (2024). “I Thought It Was Better to Be Safe Than Sorry”: Factors Influencing Parental Decisions on HPV and Other Adolescent Vaccinations for Students with Intellectual Disability and/or Autism in New South Wales, Australia. Vaccines.

[28] Tuckerman J., Mohamed Y., Justice F., Andersson T., Wyatt K., Broun K., Bastable A., Overmars I., Kaufman J., Danchin M. (2024). Stakeholder Perspectives of Immunisation Delivery for Adolescents with Disability in Specialist Schools in Victoria, Australia: ‘We Need a Vaccination Pathway’. BMC Public Health.

[29] Baines S., Emerson E., Robertson J., Hatton C. (2018). Sexual Activity and Sexual Health among Young Adults with and without Mild/Moderate Intellectual Disability. BMC Public Health.

[30] Verbeek M.C., Luijk M., Weeland J., van de Bongardt D. (2023). Male Adolescents with Mild Intellectual Disabilities: Normative Sexual Development and Factors Associated with Sexual Risks. Sex. Disabil..

[31] Estruch-García V., Gil-Llario M.D., Fernández-García O. (2024). Sexual Experiences and Knowledge of People with Moderate Intellectual Disability. J. Intellect. Disabil. Res..

[32] Vivet N., de La Rochebrochard E., Martin P. (2025). Young People with Disabilities and Their Sexual Health: A Descriptive Review of Needs, Recommendations and Interventions. BMC Public Health.

[33] Wos K., Kamecka-Antczak C., Szafrański M. (2021). In Search of Solutions Regarding the Sex Education of People with Intellectual Disabilities in Poland—Participatory Action Research. Eur. J. Spec. Needs Educ..

[34] Graff H.J., Moyher R.E., Bair J., Foster C., Gorden M.E., Clem J. (2018). Relationships and Sexuality: How Is a Young Adult with an Intellectual Disability Supposed to Navigate?. Sex. Disabil..

[35] Rooks-Ellis D.L., Jones B., Sulinski E., Howorth S., Achey N. (2020). The Effectiveness of a Brief Sexuality Education Intervention for Parents of Children with Intellectual and Developmental Disabilities. Am. J. Sex. Educ..

[36] Pozniak K., King G., Chambers E., Martens R., Earl S., Kraus de Camargo O., McCauley D., Teplicky R., Rosenbaum P. (2024). What Do Parents Want from Healthcare Services? Reports of Parents’ Experiences with Pediatric Service Delivery for Their Children with Disabilities. Disabil. Rehabil..

[37] United Nations (2006). Convention on the Rights of Persons with Disabilities.

[38] Adams R.C., Levy S.E. (2017). Shared Decision-Making and Children with Disabilities: Pathways to Consensus. Pediatrics.

[39] Downs J., Keeley J., Skoss R., Mills J., Nevill T., Schippers A., Lindly O., Thompson S. (2024). Perspectives on the Essential Skills of Healthcare Decision Making in Children and Adolescents with Intellectual Disability. Int. J. Equity Health.

[40] Gudelytė U., Ruškus J., McCrea K.T. (2024). “Help Me to Decide”: A Study of Human Rights-Based Supported Decision Making with Persons with Intellectual Disabilities. Am. J. Orthopsychiatry.

[41] Demic S., van den Breemer R., Hanisch H., Lid I.M. (2025). Care and Choice Architecture: Relatives’ Support for Adults with Intellectual Disabilities in Supported Decision-Making Processes. J. Intellect. Disabil..

[42] Casey H., Trayer Á., Desmond D., Coffey L. (2025). Experiences and Perceptions of Everyday Decision-Making in the Lives of Adults with Intellectual Disabilities, Their Care Partners and Direct Care Support Workers. J. Intellect. Disabil..

[43] Duran C., Gupta A., Aririguzo L., Castillo N., Misra S.M. (2025). Review of Human Papillomavirus Vaccination Programs in United States Schools. Vaccines.

[44] Dussault J.M., Rivera Torres A.F., Oudin Doglioni D., Gagneux-Brunon A., Le Duc-Banaszuk A.-S., Bruel S., Michel M., Gauchet A., Barret A.-S., Sicsic J. (2025). The Effect of School-Based Interventions on HPV Vaccine-Related Knowledge, Attitudes, and Practices among Adolescents in France: Secondary Results from the PrevHPV Trial. Vaccine.

[45] Thilly N., Michel M., Simon M., Bocquier A., Gagneux-Brunon A., Gauchet A., Gilberg S., Le Duc-Banaszuk A.-S., Bruel S., Mueller J.E. (2024). Effectiveness of a School- and Primary Care–Based HPV Vaccination Intervention. JAMA Netw. Open.

[46] Klinner C., McCormack B., Young A., Newman C.E., Szanto T., Brogan D., Carter A. (2026). Improving School Vaccinations for Adolescents with Intellectual and Developmental Disabilities: A Person-Centred Approach. J. Adv. Nurs..

