# Supplementary material for: Knowledge, Attitudes, and Perceptions of HPV Vaccination for Adolescents with Intellectual Disabilities: A Systematic Review
Source: Vaccines (Basel). 2026 Sep 1;14(9):764. doi: 10.3390/vaccines14090764 (PMC13611418; doi:10.3390/vaccines14090764)
Supplement: Supplementary file 1 [file vaccines-14-00764-s001.zip › vaccines-4516568-supplementary.pdf]

SUPPLEMENTARY FILE

Systematic review

Knowledge, attitudes, and perceptions of HPV vaccination for adolescents with intellectual disabilities: a systematic review

Giuseppina Lo Moro[1], Erica De Vita[2*] and Gianluca Voglino[3]

[1] Department of Public Health and Pediatric Sciences, University of Turin, 10126, Turin, Italy; giuseppina.lomoro@unito.it
[2] Department of Translational Research and New Technologies in Medicine, University of Pisa, 56100, Pisa, Italy; erica.devita@unipi.it
[3] Ministry of Health, 00144, Rome; gianluca.voglino@gmail.com
This review reflects the independent work of the authors and is not conducted under the mandate of one institution.

* Correspondence: erica.devita@unipi.it.

Table of contents

S1. SEARCH STRATEGY 2

S2. RISK OF BIAS 3

S1. SEARCH STRATEGY

PUBMED:

(“mental retard*”[Title/Abstract] OR “intelligence defic*”[Title/Abstract] OR “intellectual impair*”[Title/Abstract] OR “mental barrier*”[Title/Abstract] OR “mental disorder*”[Title/Abstract] OR “mental development”[Title/Abstract] OR “intellectual development”[Title/Abstract] OR “intellectual disab*”[Title/Abstract] OR “cognitive impair*”[Title/Abstract] OR “developmental disab*”[Title/Abstract] OR “mental* handicap*”[Title/Abstract] OR “intellectual* handicap*”[Title/Abstract] OR “Down Syndrome”[Title/Abstract] OR “Williams Syndrome”[Title/Abstract] OR “Prader Willi Syndrome”[Title/Abstract] OR “Rett syndrome”[Title/Abstract] OR "Persons with Intellectual Disabilities"[Mesh] OR "Intellectual Disability"[Mesh]) AND (Vaccine*[tiab] OR Vaccination*[tiab] OR immuniz*[tiab] OR immunis*[tiab] OR “Vaccines”[Mesh] OR “Immunization”[Mesh] OR “Vaccination”[Mesh]) AND (HPV[tiab] OR papillomavirus[tiab])

SCOPUS:

TITLE-ABS-KEY(“mental retard*” OR “intellectual disab*” OR “intelligence defic*” OR “intellectual impair*” OR “mental barrier*” OR “mental disorder*” OR “intellectual development” OR “mental development” OR “cognitive impair*” OR “developmental disab*” OR “mental handicap*” OR “intellectual handicap*” OR “Down syndrome” OR “Prader Willi Syndrome” OR “Rett syndrome” OR “Williams syndrome”) AND TITLE-ABS-KEY(Vaccine* OR Vaccination* OR immuniz* OR immunis*) AND TITLE-ABS-KEY(HPV OR papillomavirus)

EMBASE:

'mental retard*':ab,ti OR 'intellectual development':ab,ti OR 'intellectual disab*':ab,ti OR 'mental disab*':ab,ti OR 'mental development':ab,ti OR 'intelligence defic*':ab,ti OR 'intelligence impair*':ab,ti OR 'mental barrier*':ab,ti OR 'mental disorder*':ab,ti OR 'cognitive impair*':ab,ti OR 'developmental disab*':ab,ti OR 'mental* handicap*':ab,ti OR 'intellectual* handicap*':ab,ti OR 'Down syndrome':ab,ti OR 'Prader Willi Syndrome':ab,ti OR 'Rett syndrome':ab,ti OR 'Williams syndrome':ab,ti

AND

vaccine*:ab,ti OR vaccination*:ab,ti OR immuniz*:ab,ti OR immunis*:ab,ti

AND

HPV:ab,ti OR papillomavirus:ab,ti

Note: The search terms were designed to identify studies related to intellectual disability. To capture all relevant literature, outdated terminology was also included. The authors do not endorse the use of these terms.

S2. RISK OF BIAS

Table S1. Qualitative studies: evaluation of risk of bias

Study	Q1.Is there congruity between the philosophical or theoretical positioning of the study and the methodological approach that has been adopted?	Q2 Is there congruity between the research methodology and the research question or objectives?	Q3. Is there congruity between the research methodology and the methods used to collect data?	Q4. Is there congruity between the research methodology and the representation and analysis of data?	Q5. Is there congruity between the research methodology and the interpretation of results?	Q6. Is there a statement locating the researcher culturally or theoretically?	Q7. Is the influence of the researcher on the research, and vice- versa, addressed?	Q8. Are participants, and where appropriate, their voices, adequately represented?	Q9. Is the research ethical according to current criteria, and for recent studies, is there evidence of ethical approval by an appropriate body?	Q10. Do the conclusions drawn in the research report flow from the analysis, or interpretation, of the data?
Bouza et al. 2021	Unclear	Yes	Yes	Yes	Yes	No	No	Yes	Yes	Yes
Carnegie et al. 2025	Yes	Yes	Yes	Yes	Yes	Yes	Yes	Yes	Yes	Yes
Carter et al. 2024	Unclear	Yes	Yes	Yes	Yes	No	Unclear	Yes	Yes	Yes
Tuckerman et al. 2024	Unclear	Yes	Yes	Yes	Yes	Unclear	Unclear	Yes	Yes	Yes

Table S2. Qualitative studies: rationale for evaluation of risk of bias

Study	Q1 Rationale	Q2 Rationale	Q3 Rationale	Q4 Rationale	Q5 Rationale	Q6 Rationale	Q7 Rationale	Q8 Rationale	Q9 Rationale	Q10 Rationale
Bouza et al. 2021	No explicit philosophical/theoretical positioning is stated. The open-ended survey plus inductive content analysis is compatible with qualitative description, but the positioning-methodology link is not made explicit.	Objective was to examine physicians' experiences/thoughts regarding HPV vaccine recommendations for females with ID; qualitative/open-ended data are appropriate for this objective.	Open-ended questions are congruent with qualitative description/content analysis, although the absence of interviewing/probing limits depth.	Data were analysed with inductive content analysis; themes/subthemes and response counts are presented, with a codebook and interrater reliability reported.	Interpretation is framed around themes emerging from physician responses and remains broadly aligned with the content-analysis approach.	No explicit statement locates the researchers culturally, theoretically, or reflexively in relation to the topic.	The influence of researchers on data generation/interpretation, or vice versa, is not discussed.	Participant characteristics are described and results include themes with illustrative quotations/physician identifiers.	Institutional Review Board approval is reported.	Conclusions about physician support, parent uncertainty/receptivity, sexual inactivity assumptions, and trauma concerns follow from reported themes.
Carnegie et al. 2025	The paper explicitly states a critical paradigm	Objectives address how young men	Activity-oriented focus groups, visual	Audio-recorded transcripts,	Interpretation focuses on discourse, exclusion	The lead researcher explicitly reflected on social position,	Power, acquiescence, deference,	Participants are described, direct extracts	Ethical approval, informed consent, anonymisation/pseu	Conclusions about inaccessible information,

	and uses critical discourse analysis/discursive psychology, which are congruent.	with ID understand, discuss, and perceive HPV/HPV vaccination; qualitative focus groups and discourse analysis are appropriate.	aids, artefacts and topic guide are congruent with inclusive/critical qualitative methodology.	analytic field notes, coding frame, discursive strategies and emerging discourses are described; team checking/refinement is reported.	from HPV information, health inequalities and accessible communication, consistent with critical discourse analysis.	gender, age, ethnicity, personal experiences, and political/professional beliefs; author backgrounds are also provided.	participant control, researcher sharing personal artefacts, and group dynamics are discussed.	are presented throughout, and analysis is linked to participants' voices and interactions.	donyms, and inclusion criteria are reported.	variable understanding of cancer/HPV and need for accessible health messages flow from the presented analysis.
Carter et al. 2024	The study states use of qualitative methodology guided by inclusive research principles, but does not clearly state a philosophical/theoretical positioning.	Aim was to explore factors influencing parental decision-making; qualitative stakeholder interviews/focus groups are congruent with this objective.	Semi-structured interviews and focus groups with multiple stakeholder groups are appropriate for exploring beliefs, concerns and decision-making contexts.	Thematic analysis using a 7-step framework, team-based coding, NVivo indexing, and independent coding of some datasets are described.	Interpretation is grounded in themes about parental positions and education/questions/concerns, consistent with thematic analysis.	No explicit researcher positionality/cultural/theoretical location is provided.	Team-based analysis and participatory workshop are described, but the influence of researchers on data collection/interpretation is not explicitly addressed.	Participant groups are described and findings include detailed quotations from parents, school staff and immunisation staff.	Ethics approval and consent procedures are reported in the paper.	Conclusions about diverse parental attitudes, safety/autism/trauma/sexual activity concerns, and communication needs follow from reported themes.
Tuckerman et al. 2024	The study explains qualitative design and reports COREQ, but does not articulate a clear philosophical/theoretical positioning.	Aim was to identify facilitators/barriers to HPV vaccination in specialist schools; qualitative interviews are congruent with exploring stakeholder experiences.	Semi-structured interviews with relevant stakeholders are congruent with the qualitative design and aims.	Inductive thematic coding by two researchers, coding framework development, regular team meetings, and mapping to UNICEF Journey to Immunization model are described.	Interpretation of before/during/after vaccination barriers and facilitators aligns with thematic approach and Journey to Immunization framework.	The paper reports interviewers' gender and qualifications, but does not fully locate researchers culturally/theoretically or reflexively.	Some details about interviewer characteristics and team discussions are reported, but researcher influence/power dynamics are not explicitly analysed.	Participants are described by stakeholder group; results include quotations and tables of barriers/facilitators and suggestions.	Ethics approvals, written consent, adolescent assent/parental consent, compensation, and withdrawal are reported.	Conclusions about accessible parent information, consent, preparation, individual needs and vaccination pathways are supported by findings.

Table S3. Quantitative studies: evaluation of risk of bias

Study	Q1 Was the sample frame appropriate to address the target population?	Q2 Were study participants sampled in an appropriate way?	Q3 Was the sample size adequate?	Q4 Were the study subjects and the setting described in detail?	Q5 Was the data analysis conducted with sufficient coverage of the identified sample?	Q6 Were valid methods used for the identification of the condition?	Q7 Was the condition measured in a standard, reliable way for all participants?	Q8 Was there appropriate statistical analysis?	Q9 Was the response rate adequate, and if not, was the low response rate managed appropriately?
Chen et al. 2024	Yes	Unclear	Unclear	Yes	Unclear	Unclear	Yes	Yes	Unclear
Cody et al 2013	Yes	Unclear	No	Yes	No	Unclear	Yes	Yes	No
Humphrey et al. 2021	Yes	Yes	Unclear	Yes	Unclear	Yes	Yes	Yes	Unclear

Table S4. Quantitative studies: rationale for evaluation of risk of bias

Study	Q1 rationale	Q2 rationale	Q3 rationale	Q4 rationale	Q5 rationale	Q6 rationale	Q7 rationale	Q8 rationale	Q9 rationale
Chen et al. 2024	The sampling frame was appropriate for the study-specific disability population: females with ASD linked to the Lurie Center/Research Patient Data Registry. It is less representative of all females with ASD, but the paper defines the cohort as clinically referred.	Potentially eligible clinic patients/guardians were identified through the registry and invited, which is appropriate for a clinic-based cohort. However, the recruitment was not population-based/random and the extent to which all eligible patients participated is not fully transparent.	The total analytic samples were moderate (e.g., 73 children for HPV vaccination, 108 adults for HPV vaccination, 82 adults for Pap smear), but no sample size calculation was reported and some subgroup analyses were small.	Participants, age groups, ASD diagnosis, setting, survey versions, demographic variables, and clinical variables including intellectual disability and language impairment were described.	Flow diagrams and exclusions were reported, but missing demographic/vaccination/screening information and duplicate/inconsistent surveys led to exclusions. Non-response patterns were not fully described.	HPV vaccination and Pap smear uptake were self-reported or proxy-reported, not verified against medical records. Barriers were based on survey options. The authors note self-report as a limitation.	The same REDCap survey approach and predefined response categories were used for participants within the child/adult survey versions.	Numerators and denominators were reported and chi-square/Wilcoxon tests were used for group comparisons. Confidence intervals for prevalence estimates were not consistently presented, but the descriptive and comparative analyses were broadly appropriate.	The response rate among all invited eligible patients was not clearly reported, and potential non-response bias was not fully assessed.
Cody et al. 2013	The sampling frame was appropriate for the study-specific disability population	Parents were recruited by convenience from clinics/flyers and providers responded to an	The parent sample was very small (n=17) and no sample size calculation	The paper describes the clinic settings, age range, disability eligibility categories, parent demographics, Child	Coverage of the identified parent sample was limited; refusal/non-participation was not documented. Provider response rate was low, limiting coverage of the sampling frame.	HPV vaccination, knowledge, attitudes and barriers were assessed with	Questionnaires were administered in a consistent way within each respondent	Analyses were descriptive and appropriate for the exploratory aims, although the small sample precluded	Provider response rate was 22%, and parent refusal/non-response was not

		online survey. This was not random or census-based sampling of the disability population.	was reported. The provider survey had more respondents, but only 77 met inclusion criteria and the response rate was low.	Function Disability Inventory distribution, and provider characteristics.		questionnaires adapted from prior surveys, but validation in parents of CSHCN was not clearly established.	group, with predefined knowledge and attitude items.	robust inferential analysis.	documented. Low response and non-response bias were not managed adequately.
Humphrey et al. 2021	The sample frame was appropriate for the study-specific disability population	All clinic patients seen during the defined period who met the Rett syndrome, sex, and age criteria appear to have been reviewed, which is appropriate for a clinic-based census sample.	The total sample included 77 participants, but outcome-specific denominators were smaller (e.g., 48 eligible with HPV vaccination data; 35 with reasons for non-receipt). No sample size calculation was reported.	The clinic setting, inclusion/exclusion criteria, age groups, Rett syndrome characteristics, mutation data, and relevant clinical characteristics were described.	The analysis excluded participants with missing data for each variable, and the extent/pattern of missingness was not fully addressed.	HPV vaccination and well-woman care outcomes were abstracted from medical records and supplemented by caregiver survey.	Data collection used chart review and supplementary survey for all participants	The study used descriptive prevalence estimates with Wilson score confidence intervals, which is appropriate for its descriptive aims.	The prospective survey response rate and non-response characteristics were not clearly reported, and missing data were handled by variable-wise exclusion.

References

Bouza, B., Hammig, B. & Schaefer Whitby, P. Physicians' Experiences of Recommending the HPV Vaccine to Females with an Intellectual Disability. Sex Disabil 39, 731–750 (2021). https://doi.org/10.1007/s11195-021-09688-x

Carnegie, E., Gray-Brunton, C., Kennedy, C., Pow, J., Willis, D., & Whittaker, A. (2025). Young men with intellectual disabilities' perceptions of HPV and HPV vaccine: A qualitative study on how to communicate HPV vaccine information. Human vaccines & immunotherapeutics, 21(1), 2491857. https://doi.org/10.1080/21645515.2025.2491857

Carter, A., Klinner, C., Young, A., Strnadová, I., Wong, H., Vujovich-Dunn, C., Newman, C. E., Davies, C., Skinner, S. R., Danchin, M., Hynes, S., & Guy, R. (2024). "I Thought It Was Better to Be Safe Than Sorry": Factors Influencing Parental Decisions on HPV and Other Adolescent Vaccinations for Students with Intellectual Disability and/or Autism in New South Wales, Australia. Vaccines, 12(8), 922. https://doi.org/10.3390/vaccines12080922

Chen, Y., Powers, J., McDougle, C. J., Zürcher, N. R., & Thom, R. P. (2024). Cervical Cancer Screening and Prevention Uptake in Females with Autism Spectrum Disorder. Journal of autism and developmental disorders, published online. https://doi.org/10.1007/s10803-024-06565-2

Cody, P. J., & Lerand, S. J. (2013). HPV vaccination in female children with special health care needs. Journal of pediatric and adolescent gynecology, 26(4), 219–223. https://doi.org/10.1016/j.jpag.2013.03.003

Humphrey, K. N., Horn, P. S., Olshavsky, L., Reebals, L., & Standridge, S. M. (2021). Well-woman care and HPV vaccination rates in women with Rett syndrome. Disability and health journal, 14(1), 100982. https://doi.org/10.1016/j.dhjo.2020.100982

Tuckerman, J., Mohamed, Y., Justice, F., Andersson, T., Wyatt, K., Broun, K., Bastable, A., Overmars, I., Kaufman, J., & Danchin, M. (2024). Stakeholder perspectives of immunisation delivery for adolescents with disability in specialist schools in Victoria, Australia: 'we need a vaccination pathway'. BMC public health, 24(1), 1973. https://doi.org/10.1186/s12889-024-19322-y
